# Proteomic fingerprints of damage in extracellular matrix assemblies

**DOI:** 10.1016/j.mbplus.2020.100027

**Published:** 2020-01-30

**Authors:** Alexander Eckersley, Matiss Ozols, Ronan O'Cualain, Emma-Jayne Keevill, April Foster, Suzanne Pilkington, David Knight, Christopher E.M. Griffiths, Rachel E.B. Watson, Michael J. Sherratt

**Affiliations:** a Division of Cell Matrix Biology & Regenerative Medicine, School of Biological Sciences, Faculty of Biology, Medicine and Health, The University of Manchester, Manchester, UK; b Division of Musculoskeletal & Dermatological Sciences, School of Biological Sciences, Faculty of Biology, Medicine and Health, The University of Manchester, Manchester, UK; c Biological Mass Spectrometry Core Research Facility, School of Biological Sciences, Faculty of Biology, Medicine and Health, The University of Manchester, Manchester, UK; d NIHR Manchester Biomedical Research Centre, Central Manchester University Hospitals NHS Foundation Trust, Manchester Academic Health Science Centre, Manchester, UK

**Keywords:** AFM, atomic force microscopy, COL6A3, collagen VI alpha 3 chain, ECM, extracellular matrix, EGF, epidermal growth factor domain, HDF, human dermal fibroblast, LC-MS/MS, liquid chromatography tandem mass spectrometry, PSM, peptide spectrum match, ROS, reactive oxygen species, SSR, solar simulated radiation, TGFβ, transforming growth factor beta, UVR, ultraviolet radiation, vWA, von Willebrand factor type A domain, Fibrillin microfibril, Collagen VI microfibril, Ultraviolet radiation, Photodamage, Mass spectrometry

## Abstract

In contrast to the dynamic intracellular environment, structural extracellular matrix (ECM) proteins with half-lives measured in decades, are susceptible to accumulating damage. Whilst conventional approaches such as histology, immunohistochemistry and mass spectrometry are able to identify age- and disease-related changes in protein abundance or distribution, these techniques are poorly suited to characterising molecular damage. We have previously shown that mass spectrometry can detect tissue-specific differences in the proteolytic susceptibility of protein regions within fibrillin-1 and collagen VI alpha-3. Here, we present a novel proteomic approach to detect damage-induced “peptide fingerprints” within complex multi-component ECM assemblies (fibrillin and collagen VI microfibrils) following their exposure to ultraviolet radiation (UVR) by broadband UVB or solar simulated radiation (SSR). These assemblies were chosen because, in chronically photoaged skin, fibrillin and collagen VI microfibril architectures are differentially susceptible to UVR. In this study, atomic force microscopy revealed that fibrillin microfibril ultrastructure was significantly altered by UVR exposure whereas the ultrastructure of collagen VI microfibrils was resistant. UVR-induced molecular damage was further characterised by proteolytic peptide generation with elastase followed by liquid chromatography tandem mass spectrometry (LC-MS/MS). Peptide mapping revealed that UVR exposure increased regional proteolytic susceptibility within the protein structures of fibrillin-1 and collagen VI alpha-3. This allowed the identification of UVR-induced molecular changes within these two key ECM assemblies. Additionally, similar changes were observed within protein regions of co-purifying, microfibril-associated receptors integrins αv and β1. This study demonstrates that LC-MS/MS mapping of peptides enables the characterisation of molecular post-translational damage (via direct irradiation and radiation-induced oxidative mechanisms) within a complex in vitro model system. This peptide fingerprinting approach reliably allows both the identification of UVR-induced molecular damage in and between proteins and the identification of specific protein domains with increased proteolytic susceptibility as a result of photo-denaturation. This has the potential to serve as a sensitive method of identifying accumulated molecular damage in vivo using conventional mass spectrometry data-sets.

## Highlights

• Mass spectrometry “peptide fingerprinting” can detect post-translational damage within extracellular matrix proteins.
• UVR-induced FBN1 and COL6A3 peptide fingerprints are reproducibly identified from purified microfibrils.
• Peptide mapping reveals increased regional susceptibilities to proteolysis in FBN1 and COL6A3 proteins.
• Regional changes are also observed in protein structures of microfibril-associated receptor integrins αv and β1.
• This “peptide fingerprinting” approach is applicable to conventional LC-MS/MS datasets.

## Introduction

In contrast to the rapid turnover which characterises intracellular proteomes [1], structural extracellular matrix (ECM) proteins are commonly long-lived, some with half-lives measured in decades [[2], [3], [4]]. As a consequence, these proteins may accumulate damage due to ageing and chronic disease [[5], [6], [7], [8]] which can manifest as changes in their abundance and architecture [[9], [10], [11]]. Although conventional histological [10,12,13], immunological [11], ultrastructural [[14], [15], [16], [17]] and mass spectrometry approaches [[18], [19], [20]] can detect tissue remodelling, these techniques are not well suited to characterising differences in molecular structure. We have previously shown that liquid chromatography tandem mass spectrometry (LC-MS/MS) analysis of purified ECM suspensions can detect tissue- and cell culture-specific peptide patterns [21] of protein structure. Here we aim to determine if the same approach can identify characteristic “peptide fingerprints” of in vitro damage (photodamage and oxidation [14,15,22]) in supramolecularly dissimilar ECM assemblies (collagen VI and fibrillin microfibrils) which are subject to contrasting fates in chronically photoaged skin [10,12].

Skin photoageing is characterised histologically by the reorganisation of the dermal ECM (see reviews: [23,24]) in response to chronic exposure to ultraviolet radiation (UVR) [25]. The elastic fibre network, and in particular the fibrillin microfibril component [26], is a sensitive indicator of both photoageing [10,27] and repair [28]. Photoageing can be characterised immunohistochemically and histologically by the loss of fibrillin microfibrils at the papillary dermis [10]. However, the presence of cumulative molecular damage within elastic fibre components, which may impair structural and biochemical functionality [29,30], has yet to be demonstrated.

Ultraviolet radiation, which reaches the Earth's surface, consists of approximately 5% UVB (wavelength 280–315 nm) and 95% longer wavelength (315–400 nm) UVA [31]. The absorption of these photons by photosensitive biomolecules can cause photochemical reactions in amino acid residues [32] leading to the direct photo-denaturation of protein (e.g. via the dissolution of di-sulphide bonds [33] or creation of new crosslinks [34]). This absorption may also lead to the photodynamic formation of reactive oxygen species (ROS) [22,35,36]. In addition to photo-denaturation, photo-induced cleavage of peptide bonds may occur theoretically however, to our knowledge, this has never been shown. Furthermore, we have previously shown that denaturation is likely the main mechanism of damage to protein structure, not fragmentation (Fig. S1) [15]. The relative susceptibility of structural proteins to UVR (characterised ultrastructurally or by electrophoretic mobility) correlates positively with the abundance of disulphide-bonded cysteine, tryptophan and tyrosine amino acid residues which act as chromophores [14,15]. Compared with most other ECM proteins, fibrillin microfibril-associated proteins (fibrillins, fibronectin, fibulins and latent transforming growth factor β binding proteins) are highly enriched in ROS- and UVR-sensitive amino acid residues [14,15]. The assembled fibrillin microfibril is a large macromolecular assembly which resembles “beads on a string” with a characteristic ~56 nm periodicity [14,15,21,[37], [38], [39], [40]]. Previously, we have shown that low-dose UVR irradiation by either broadband UVB [15] or solar simulated radiation (SSR: 95% UVA + 5% UVB) [14,38] causes quantifiable changes to fibrillin microfibril ultrastructure. It is likely, therefore, that the degeneration of the fibrillin microfibril network observed histologically during photoageing [10] may be due, in part, to an accumulation of molecular damage and potentially an increased susceptibility to protease-mediated digestion [22]. In contrast, the constituent alpha chains of collagen VI microfibrils contain fewer UVR chromophore amino acid residues [14] and the assembled double-beaded microfibrils are resistant to both architectural remodelling in photoaged skin [12] and structural remodelling following UVR exposure in vitro [14]. In common with fibrillin microfibrils, collagen VI microfibrils also play important biochemical roles: anchoring cells to the matrix [41] and interacting with numerous ECM components [42,43]. As the constituent collagen VI proteins are not completely devoid of UVR- and ROS-sensitive amino acid residues, these abundant skin components may accumulate molecular damage which is not detectable immunohistochemically or ultrastructurally.

Molecular damage may be detected by conventional biochemical techniques such as gel electrophoresis; however, the high molecular weights and insolubility of these macromolecular ECM assemblies makes this approach challenging [39,40]. Recently, we have demonstrated that mass spectrometry-based proteomics can be used to effectively detect regional and compositional differences in the susceptibility of fibrillin and collagen VI microfibrils to elastase digestion [21]. By mapping LC-MS/MS detected peptide spectrum matches (PSMs) of fibrillin-1 and collagen VI alpha-3 (COL6A3) to their corresponding domain structures, we demonstrated tissue and cell culture-specific local differences in structure within these assemblies, on a molecular scale. In this study, we aimed to use these methods [21] to determine if in vitro broadband UVB (0.1 J/cm^2^) and SSR (30 J/cm^2^) exposure induced characteristic “peptide fingerprints” of molecular damage in co-purified suspensions of fibrillin and collagen VI microfibrils, derived from human dermal fibroblasts (HDFs). This approach has the potential to identify molecular damage in ECM proteins in skin and other ageing and diseased tissues.

## Results and discussion

### Fibrillin microfibril ultrastructure is susceptible to both broadband UVB and solar simulated radiation

**Fig. 1:**
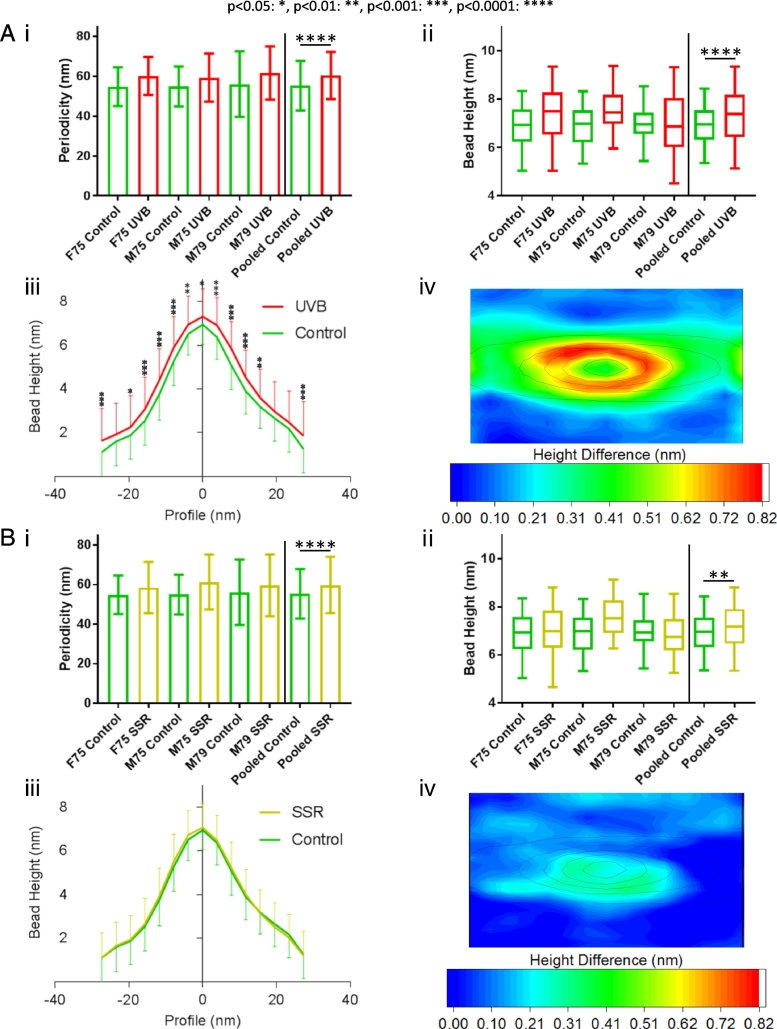
HDF-derived fibrillin microfibril ultrastructure was affected by both broadband UVB and SSR. UVB-irradiated (0.1 J/cm^2^) fibrillin microfibril periodicities were significantly higher (n = 500 repeats, n = 1500 pooled) than those of control (p < 0.0001, Mann-Whitney U) (Ai; data = mean and SD). Central bead heights were also significantly higher in UVB-irradiated fibrillin microfibrils (n = 100 repeats, n = 300 repeats pooled) than in control (p < 0.0001, Mann-Whitney U) (Aii; data = median, IQR and range). The axial profiles of UVB-irradiated microfibril beads were also significantly higher along the entire central bead axis than those of control (Bonferroni multiple comparison test) (Aiii; data = mean and SD). In order to visualise UVR-induced changes in bead morphology, AFM height maps of control microfibril beads were averaged and subtracted from that of UVB-irradiated microfibril beads. UVB-induced changes in morphology can be observed throughout the bead but primarily along its slopes (~10 nm radius from the peak) (Aiv). Fibrillin microfibril ultrastructure was less affected by SSR (30 J/cm^2^; B). SSR-irradiated microfibril periodicities were significantly higher than those of control (p < 0.0001, Mann-Whitney U) (Bi; data = mean and SD). Central bead heights were also significantly higher in SSR-irradiated fibrillin microfibrils compared to control (p = 0.0061, Mann-Whitney U) (Bii; data = median, IQR and range). However, axial profiles of SSR-irradiated fibrillin microfibril beads did not differ significantly from those of control (Bonferroni multiple comparison test) (Biii; data = mean and SD). Heat mapped height differences between SSR-irradiated and control show only small changes in bead morphology near central peak and along one of the lateral slopes (Biv).

Prior to LC-MS/MS analysis, broadband UVB- and SSR-induced damage to fibrillin and collagen VI microfibrils was assessed using atomic force microscopy (AFM). Both broadband UVB and SSR caused quantifiable changes to fibrillin microfibril ultrastructure. Broadband UVB irradiation induced significant increases in fibrillin microfibril periodicity (Fig. 1 Ai) and central bead height (Fig. 1 Aii), as well as significant changes in bead morphology compared to non-irradiated microfibrils. According to axial bead height profiles, broadband UVB-irradiated fibrillin microfibril bead height was significantly greater along the entire repeat profile when compared to the bead height of non-irradiated fibrillin microfibrils (Fig. 1 Aiii). Contour heat mapping of bead height differences also demonstrated that broadband UVB irradiation caused beads to increase in volume (Fig. 1 Aiv). The lower energy wavelengths in SSR were less damaging to fibrillin microfibril ultrastructure; SSR caused significant increases in both fibrillin microfibril periodicity (Fig. 1 Bi) and central bead height (Fig. 1 Bii), but induced only minimal changes in the axial height profile (Fig. 1 Biii). However, contour heat mapping of microfibril repeats indicated measurable differences close to the bead centre (Fig. 1 Biv).

The changes in fibrillin microfibril periodicity seen in response to both SSR and broadband UVB irradiation are consistent with previous studies [14,15,38]. However, changes to bead morphology using AFM height mapping have not previously been demonstrated. Using scanning transmission electron microscopy, we reported previously that broadband UVB caused a loss of mass within the beads of both skin- and cell-derived fibrillin microfibrils [15]. Our AFM height mapping, however, showed an increase in bead volume. Collectively these observations suggest that exposure to UVR induced structural changes which result in a loss or re-organisation of microfibrillar proteins (either from fibrillin-1 or associated proteins) and, consequentially a reduced packing density [39,[44], [45], [46], [47]] and increased bead volume.

### Collagen VI microfibril ultrastructure is largely resistant to SSR- and UVB-induced damage

**Fig. 2:**
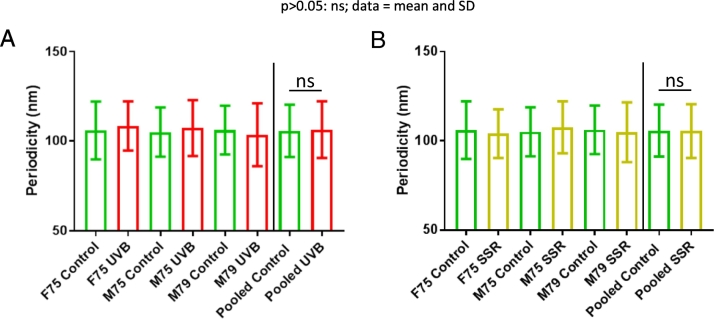
Broadband UVB and SSR irradiation of HDF-derived microfibril isolations did not induce changes to collagen VI microfibril periodicity. Periodicities of both broadband UVB-irradiated (A) and SSR-irradiated (B) collagen VI microfibrils (n = 500 repeats, n = 1500 repeats pooled) were not significantly different compared those of control (UVB: p = 0.1811, SSR: p = 0.9778; Mann-Whitney U).

Atomic force microscopy quantification showed that collagen VI microfibril ultrastructure was resistant to remodelling by both broadband UVB and SSR radiation (Fig. 2). Neither broadband UVB (Fig. 2 A) nor SSR (Fig. 2 B) was able to cause measurable changes to collagen VI microfibril periodicity. Although we have previously shown that collagen VI microfibril ultrastructure is resistant to SSR, in contrast to fibrillin microfibrils [14], here we demonstrate that these assemblies were as resistant to higher energy broadband UVB as they were to SSR. Since, in skin, collagen VI microfibrils are predominantly distributed in the upper papillary dermis, near the dermal-epidermal junction [12], they are likely subjected in vivo to both UVA and UVB wavebands [14]. Therefore, their resistance to UVR may be inherent to their fundamental function and may explain the lack of change observed in collagen VI microfibril distribution in photoaged versus photoprotected skin [12].

Although collagen VI microfibril ultrastructure and architecture appeared resistant to UVR in vitro [14] and in vivo [12], molecular-scale changes may still be present which AFM and histology are unable to resolve. By measuring the propensity of these UVR-exposed collagen VI and fibrillin microfibrils to proteolytically yield fibrillin-1 and COL6A3 peptides, via elastase digestion followed by LC-MS/MS, we assessed whether damage exists on a molecular scale [21].

### UVR exposure enhances the elastase-mediated detection and yield of fibrillin-1 peptides

**Fig. 3:**
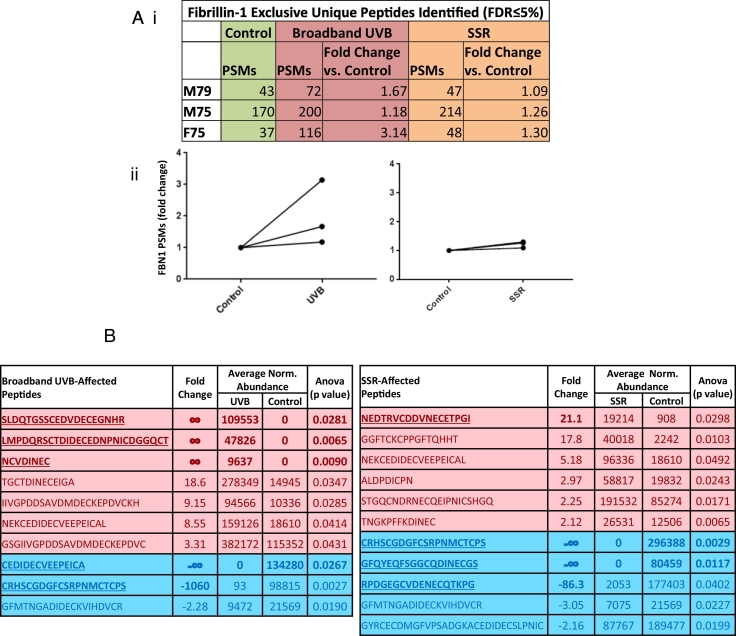
UV irradiation of HDF-derived microfibril isolations led to an increase in the overall proteolytic susceptibility of fibrillin-1. PSMs for broadband UVB- and SSR-irradiated fibrillin-1 per individual and their fold changes in comparison to control fibrillin-1 are shown (Peptide Prophet FDR ≤ 5%) (Ai). Broadband UVB irradiation led to an increase in fibrillin-1 peptide identification (2.00 average fold change) (Aii, left panel). Similarly, SSR irradiation of fibrillin-1 led to a smaller but consistent increase in fibrillin-1 peptide identification (1.22 average fold change) for all three replicates (Aii, right panel). Data-dependent peptide quantification revealed significant changes in the relative abundance of fibrillin-1 peptide sequences post-UVR exposure. Only peptides with fold changes greater than or equal to two were considered (B; N = 3). Ten fibrillin-1 peptides in broadband UVB-irradiated microfibril samples and eleven in SSR-irradiated samples were significantly increased (red) or decreased (blue) in relative abundance compared to control samples. Peptide sequences, fold changes relative to the control group, average normalised abundances and p values are shown (Progenesis multivariate paired ANOVA). Peptide sequences with fold changes greater than twenty are in bold and underlined.

Total numbers of LC-MS/MS-detected fibrillin-1 PSMs were consistently higher for broadband UVB-irradiated and SSR-irradiated fibrillin microfibril samples than for unirradiated samples (Fig. 3 Ai). Broadband UVB irradiation led to a mean increase of 55% in the number of fibrillin-1 PSMs compared to control (irradiated, 129 vs. unirradiated, 83) (Fig. 3 Aii left panel) whereas SSR irradiation led to a mean increase of 24% (irradiated, 103 vs. unirradiated, 83) (Fig. 3 Aii right panel). This increase in fibrillin-1 PSMs from irradiated microfibril samples is likely due to the changes in fibrillin microfibril ultrastructure observed using AFM (Fig. 1). The ultrastructural changes in microfibril periodicity and bead volume caused by UVR increase the accessibility of elastase to previously buried (cryptic) cleavage sites, leading to an increase in peptide yield. This yield correlated with the energy of the incident radiation: high energy broadband-UVB induced more ultrastructural damage (Fig. 1) and increased the proteolytic susceptibility of fibrillin-1 (Fig. 3 A) than lower energy SSR.

In addition to PSM numbers, data-dependent quantification revealed a number of fibrillin-1 peptides which were significantly different in relative abundance in broadband UVB-irradiated or SSR-irradiated compared to non-irradiated fibrillin microfibril samples (Fig. 3 B). Notably, both UVB and SSR irradiation led to the detection of peptide sequences (five for UVB, four for SSR), with fold changes greater than twenty compared to unirradiated control samples (Fig. 3 B, bold and underlined text). These large significant changes in relative abundance collectively suggest that UVR-induced remodelling of the tertiary and quaternary structure of fibrillin microfibrils (observed by AFM, Fig. 1) can both mask and unmask fibrillin-1 sequences, altering their accessibility to proteolytic cleavage and hence their detectability by LC-MS/MS.

### UVR exposure of collagen VI microfibrils has no effect on total collagen VI alpha-3 peptide yield but does liberate SSR- and broadband UVB-induced peptides

**Fig. 4:**
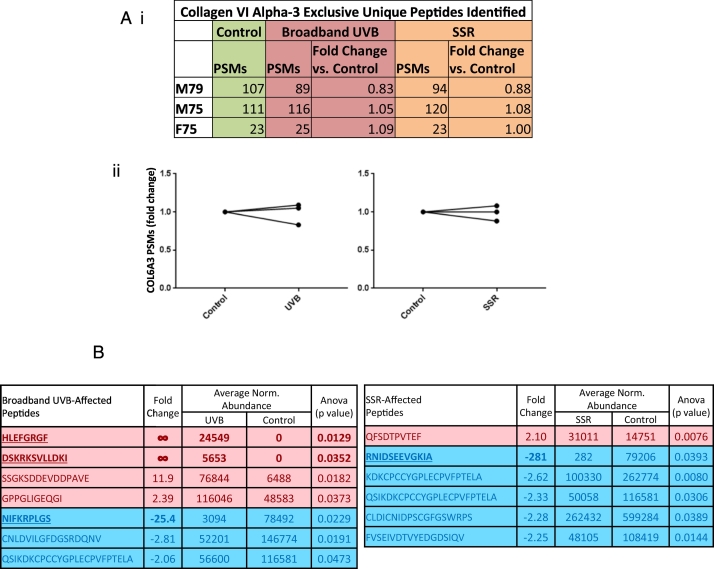
UV irradiation of HDF-derived microfibril isolations did not cause changes to the overall proteolytic susceptibility of COL6A3. PSMs for broadband UVB- and SSR -irradiated COL6A3 per individual and their fold changes in comparison to control COL6A3 are shown (Peptide Prophet FDR ≤ 5%) (Ai). In contrast to fibrillin-1, both broadband UVB- (Aii, left panel) and SSR-irradiation (Aii, right panel) failed to cause any consistent change in the number of COL6A3 peptides identified (average change = 0.99 for both UVB and SSR). However, data-dependent peptide quantification did reveal significant changes in the relative abundance of COL6A3 peptide sequences post-UVR exposure. Only peptides with fold changes greater than or equal to two were considered (B; N = 3). Seven COL6A3 peptides in broadband UVB-irradiated microfibril samples and six in SSR-irradiated samples were significantly increased (red) or decreased (blue) in relative abundance compared to control samples. Peptide sequences, fold changes relative to the control group, average normalised abundances and p values are shown (Progenesis multivariate paired ANOVA). Peptide sequences with fold changes greater than twenty are in bold and underlined.

In contrast to fibrillin-1, total numbers of LC-MS/MS-detected COL6A3 PSMs (Fig. 4 Ai) changed negligibly for broadband UVB- (Fig. 4 Aii left panel) and SSR-irradiated (Fig. 4 Aii right panel) microfibril samples compared to unirradiated samples (average 1% decrease for both SSR and broadband UVB). Therefore, in vitro UVR exposure has minimal impact on collagen VI ultrastructure (Fig. 2) and overall COL6A3 protease susceptibility (Fig. 4 A) whilst chronic in vivo exposure to UVR does not affect collagen VI architecture in human dermis [12]. We have suggested previously that this relative UVR resistance may be due to the relatively low abundance of UVR chromophore amino acids in collagen VI alpha chains compared to fibrillin-1 [14]. However, as with fibrillin-1, data-dependent quantification revealed a number of COL6A3 peptides which were significantly different in relative abundance in both broadband UVB- and SSR-irradiated microfibril samples compared to unirradiated (Fig. 4 B). Once again, both UVB and SSR irradiation led to the detection of peptide sequences (three for UVB, one for SSR), with fold changes greater than twenty compared to unirradiated samples (Fig. 4 B, bold and underlined text).

Although a number of significantly different COL6A3 peptides were affected by both SSR and broadband UVB (Fig. 4 B), only five (four for UVB and only one for SSR) had fold changes ≥±5 compared to the 14 identified from fibrillin-1 (Fig. 3 B) (eight for UVB and six for SSR). Fibrillin-1 also yielded six peptides with fold changes of infinity (four for UVB and two for SSR) compared to COL6A3 which yielded two (only in UVB). These observations collectively suggest that, with fibrillin-1, more UVR-induced changes are detected than with COL6A3, once again indicating a divergence of UVR susceptibility as noted previously, ultrastructurally [14] and histologically [12]; however this time on a molecular scale.

The use of peptide degradation products in the detection of disease is not a novel concept: oncopeptidomics is a growing field which attempts to use the LC-MS/MS detection of peptide biomarkers as a diagnostic tool for tumour presence [48,49] (see review: [50]). Additionally, the LC-MS/MS detection of peptide degradation products has been used to identify biomarkers of Fanconi syndrome [51], inflammation [52] and even allergy [53]. Although these approaches focus on the detection of single peptides as biomarkers of disease, our approach instead enables the identification of peptides which together reveal the degree of post-translational damage within the structures of abundant ECM proteins, previously undetectable using conventional methods, which could potentially impact on their function in tissue.

### Fibrillin-1 and collagen VI alpha-3 protein structures exhibit UVR-specific regional foci of proteolytic susceptibility

**Fig. 5:**
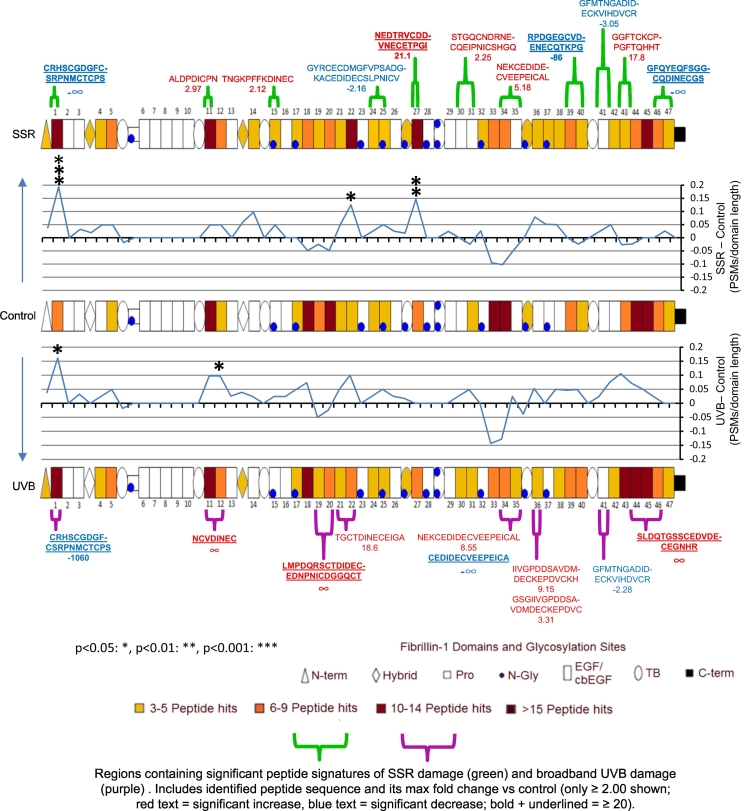
SSR and broadband UVB irradiation of HDF-derived microfibril isolations leads to changes in the proteolytic susceptibility of specific protein regions within fibrillin-1. LC-MS/MS-identified fibrillin-1 peptide sequences (PSMs: peptide prophet FDR ≤ 5%) were counted for each respective protein domain, normalised based on total spectrum count, averaged (N = 3) and subsequently heat mapped per group. Only domains containing an average of three peptides or more are shown. The PSM number corresponding to each broadband UVB- and SSR-irradiated fibrillin-1 domain were then subtracted from the counts of control and divided by the domain's primary sequence length to show regional fluctuations in proteolytic susceptibility (line graphs). Domains exhibiting significant differences in PSM numbers are also indicated (Bonferroni-corrected multiple comparisons tests taken from Fig. S2). Data-dependently quantified peptide sequences which were significantly different in relative abundance (taken from Fig. 3 B) are also mapped alongside their fold changes. UV-induced damage to fibrillin-1 is spread throughout the structure, although the N-terminal region (EGF 1) and EGFs 12, 22, 27, 33–34 and 43–45 appear the most affected. The pattern of changes seen throughout the fibrillin-1 structure is consistent in many regions, regardless of the UV irradiation source used (SSR or broadband UVB).

This study is not first to show that UVR exposure can increase the susceptibility of an ECM assembly to proteolysis; Menter et al. (2003) previously demonstrated that the UV irradiation of murine collagen I significantly increased its susceptibility to proteolysis by bacterial collagenase [54]. Menter et al. (2003) went on to suggest that the regional susceptibility of collagen I to UVR may be dependent on its superstructure and the supramolecular organisation of its protein components [54]. In an attempt to identify this in our microfibril assemblies, fibrillin-1 PSMs and significantly different, data-dependently quantified peptides from SSR-irradiated and broadband UVB-irradiated samples were mapped to the protein domain structure and compared (Fig. 5 and Fig. S2). Mapped differences in fibrillin-1 PSMs per domain, compared to control, were remarkably consistent between fibrillin microfibrils separately irradiated with either SSR or broadband UVB, with many of the same patterns observed. The largest regional differences, consistent within both SSR- and broadband UVB-irradiated fibrillin-1, can be seen near the N-terminal domain at epidermal growth factor-like (EGF) domain 1 (significantly different for both SSR and UVB; Fig. 5 and Fig. S2) and within EGF22 (significantly different for SSR). Both domains exhibited increases in PSM numbers, in contrast to EGFs 33 and 34 which exhibited decreases in PSM numbers both post UVB and SSR irradiation. Encouragingly, many of the data-dependently quantified peptides with significant differences in abundance coincide with regions exhibiting the largest differences in PSMs compared to control (Fig. 5). This is especially the case for peptide sequences with fold changes greater than or equal to twenty (bold and underlined) which tend to fall on the same domains containing significant differences in PSM numbers (stars; seen at EGF 1 for both SSR and UVB, EGF 27 for SSR and EGF12 for UVB). As shown previously, the fluctuation in peptide counts within the different regions of fibrillin-1 are indicative of the propensity of elastase to yield peptides specifically at these molecular locations [21]. The changes seen here are most likely directly related to the disruption of protein folding within the tertiary structure, via the dissolution of bonds by direct UVR or indirect photodynamic oxidation [14,22,35]. This is evidenced further by the direct changes seen in fibrillin microfibril ultrastructure (Fig. 1). Additionally, a large region of increased PSM counts can be seen exclusively in broadband UVB-irradiated fibrillin-1 compared to control, and not in SSR-irradiated; close to the C-terminus, between EGFs 42–45; (Fig. 5 and Fig. S2). This could be related to the greater damage caused by broadband UVB to the fibrillin microfibril ultrastructure compared to SSR.

**Fig. 6:**
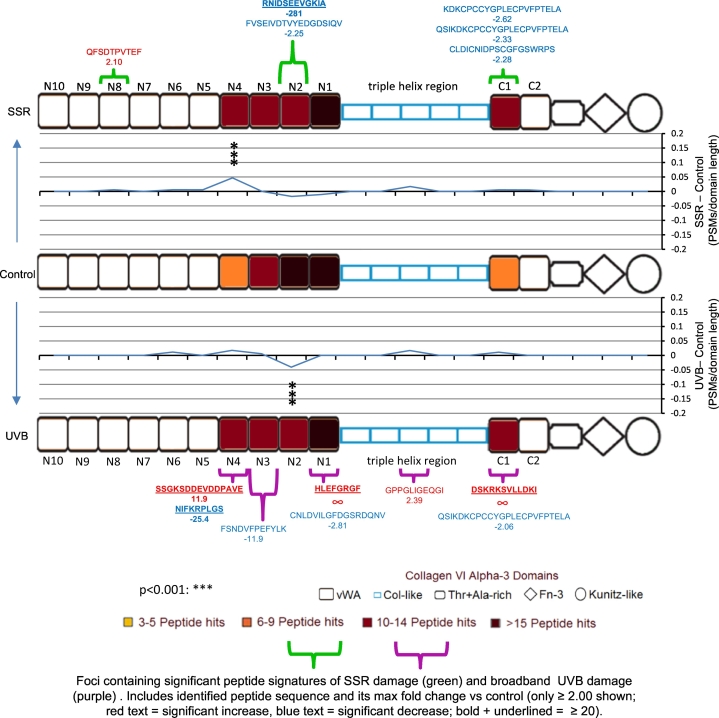
SSR and broadband UVB irradiation of HDF-derived microfibril isolations also leads to changes in the proteolytic susceptibility of specific protein regions within COL6A3. LC-MS/MS identified COL6A3 peptide sequences (PSMs: peptide prophet FDR ≤ 5%) were counted for each respective protein domain, normalised based on total spectrum count, averaged (N = 3) and subsequently heat mapped. Only domains containing an average of three peptides or more are shown. The PSM number corresponding to each broadband UVB- and SSR-irradiated COL6A3 domain were then subtracted from the counts of control and divided by the domain's primary sequence length to show regional fluctuations in proteolytic susceptibility (line graphs). Domains exhibiting significant differences in PSM numbers are also indicated (Bonferroni-corrected multiple comparisons tests taken from Fig. S3). Significantly different peptide sequences taken from Fig. 4 B and their fold changes are also mapped. Regional UVR damage to COL6A3 was limited compared to fibrillin-1, the highest (significant) changes in PSMs/domain length were seen within N4 (vWA) domain for SSR-irradiated and N2 domain for broadband UVB-irradiated. For both SSR and broadband UVB, data dependently quantified peptide sequences (which were significantly different in relative abundance) appear to cluster at vWA domains on the triple helix side of both globular regions of COL6A3: between N1 and N4 and at C1.

As with fibrillin-1, COL6A3 PSMs and significantly different data-dependently quantified peptides from SSR-irradiated and broadband UVB-irradiated microfibril samples were also mapped to the protein domain structure and compared (Fig. 6). Consistent with the previous UVR-resistance seen in collagen VI microfibril ultrastructure compared to fibrillin-1 [14], COL6A3 (Fig. 6) had lower regional fluctuations in PSM number compared to fibrillin-1 (Fig. 5), on the same PSMs/domain length scale (line graph). However, significant regional changes in COL6A3 PSM numbers were observed at von Willebrand factor type A (vWA) domains N2 in UVB-irradiated samples and N4 in SSR-irradiated (Fig. 6 and Fig. S3). Although periodicity measurements indicated no significant differences in collagen VI microfibril ultrastructure as a consequence of UVR irradiation (Fig. 2), this proteomic analysis revealed regional foci of increased proteolytic susceptibility within the protein structure of COL6A3, in response to SSR and UVB radiation.

The identification of UVR-induced regional changes to fibrillin-1 and COL6A3s demonstrates the capability and potential of LC-MS/MS “peptide fingerprinting” (the mapping and relative quantification of peptide numbers and intensities within protein domains or regions) for the detection of molecular damage within key dermal ECM assemblies. Additionally, the higher degree of change seen within the fibrillin-1 structure (Fig. 5) compared to COL6A3 (Fig. 6) correlates with the scale of ultrastructural damage observed in the microfibrils by AFM (Fig. 1, Fig. 2), as well as their chromophore amino acid content [14]. This indicates that peptide fingerprinting can also be used to assess the severity of damage within ECM assemblies. Finally, peptide fingerprinting provides a more sensitive method for detecting UVR damage than ultrastructure (Fig. 2) [14], since UVR-induced changes in COL6A3 protein structure were identified in both broadband UVB- and SSR-irradiated microfibril isolations (Fig. 6). To summarise, this peptide fingerprinting approach reliably enables both the identification of UVR-induced molecular damage in and between proteins and the identification of specific protein domains with increased proteolytic susceptibility as a result of photo-denaturation.

Significant foci of UVR-enhanced proteolytic susceptibility were observed at fibrillin-1 EGF domains 1 (for SSR and UVB), 12 (for UVB only), 22 and 27 (for SSR only; Fig. 5). Should UVR exposure promote increased enzyme cleavage of these domains by endogenous proteases in vivo, it may have downstream consequences to fibrillin-1 function in photoexposed tissue [55]. EGF 1 is located near the N-terminal region of fibrillin-1 which is known to bind a number of known fibrillin microfibril-associated proteins [55,56]. These include the homeostatic cytokines TGFβ [29,[57], [58], [59]] and BMPs [30,60,61], the elastic fibre protein MAGP1 [62,63], fibulins 2, 4 and 5, [[64], [65], [66]], and ADAMTSs 10 and 6 [67]. EGF 12 is located further along the N-terminal half of fibrillin-1 which is known to interact with the glycoprotein perlecan [68]. Finally, EGFs 22 and 27 are located in the medial region of the protein which is known to bind the key elastic fibre protein tropoelastin, as well as proteoglycans versican [69], brevican, aggrecan and neurocan [70]. Due to these many interactions, there is a distinct possibility that the increased proteolysis of these domains, as a result of accumulating UVR damage, may compromise the ability of the fibrillin microfibril to functionally interact with these key tissue modulators in vivo. The age-dependent accumulation of UVR-induced damage to fibrillin-1 may consequently affect elastogenesis and skin homeostasis and function over time.

In addition to the direct accumulation of damage in ECM assemblies by UVR and ROS, another proposed mechanism of photoageing is that exposure of keratinocytes and fibroblasts to UVR can lead to an increase in the expression of ECM proteases such as the matrix metalloproteases (MMPs) [[71], [72], [73], [74]]. The consequential increase in protease activity within the skin dermis is thought to promote the degradation of these ECM assemblies via proteolysis [22,75,76]. This study demonstrates that UVR-induced changes to fibrillin-1 and COL6A3 structure within the microfibril increases their regional susceptibility to enzymatic digestion. We postulate that the UVR-mediated increase in protease susceptibility of ECM proteins may be one of the mechanisms of photoageing which leads to the architectural degeneration seen histologically in their resident assemblies such as elastic fibres [10,77,78], collagens [9,[79], [80], [81]] and their associated proteins [11,82,83].

The increased susceptibility of UVR-exposed ECM proteins to proteolysis may lead to the aberrant release of peptides in vivo, similar to the processes seen in this study (Figs. 3B and 4B). Matrikines are described as peptides released through the proteolysis of ECM macromolecules, which are able to modulate cell activities such as proliferation, migration and apoptosis [84] through their proposed interactions with multiple cell receptors such as the EGF receptors [85] and integrins [86]. As a result, these matrikines can play a significant role is tissue homeostasis [87]. This study indicates that chronic UVR exposure of ECM macromolecules in skin, over time, may lead to the sustained, aberrant proteolytic release of matrikines from photo-denatured proteins [88]. In turn, the resultant increased production of matrikines would likely have consequences to tissue homeostasis [89] with age-specific implications in inflammation [90], tumorigenesis [91] and fibrosis [92].

### Differences in fibrillin-1 and collagen VI alpha-3 chromophore content may account for an overall divergence in UVR susceptibility

Three amino acids have the ability to absorb UVB and UVA wavebands: tryptophan, tyrosine and double-bonded cysteine (cystine) [32]. This has led to the hypothesis that these chromophores are most responsible for the direct photo-denaturation of proteins in UVR-exposed tissues. In previous studies, we showed that the UVR susceptibility of a dermal ECM assembly correlates with its total chromophore content (% of these three amino acids) within four different assemblies: fibrillin microfibrils (fibrillin-1: 16.4%), fibronectin (fibronectin-1: 8.54%), type I collagen (α1 and α2 chains combined: 0.32%) and collagen VI microfibrils (α1, α2 and α3 chains combined: 3.66%) [14,15].

The majority of fibrillin-1 domains have a chromophore content of above 7.5% with an average of ~16%; in contrast, all COL6A3 domains (with the exception of the Kunitz-like domain [93]) have a chromophore content below 7.5% with an average of ~3%. This likely indicates that the UVR susceptibility of fibrillin-1 is much greater than that of COL6A3, and may explain why the overall damage to fibrillin-1 seen by LC-MS/MS (Fig. 3 A) was higher than that of COL6A3 (Fig. 4 A). The effect of this susceptibility would likely have caused the higher ultrastructural damage seen to the fibrillin microfibrils (Fig. 1) compared to collagen VI microfibrils (Fig. 2). However, the regional changes in proteolytic susceptibility within the structures of fibrillin-1 (Fig. 5) and COL6A3 (Fig. 6), as a result of UVR exposure, do not correlate with the even distribution of chromophores. This is because peptide fingerprinting measures UVR-mediated changes in the accessibility of different proteinaceous regions to the peptide-generating elastase rather than direct changes to protein structure by UVR. This means that any regional change in peptide numbers would correlate to UVR-induced changes in the higher order tertiary and quaternary structure of these proteins within their respective microfibrils, rather than that of their primary structures.

### Microfibril-associated receptor proteins may also exhibit UVR-specific regional foci of proteolytic susceptibility

As ECM assemblies, fibrillin and collagen VI microfibrils network with a host of proteins within the dermis [55,94]. Recently, we characterised the interactors of these microfibril species isolated from eye (ciliary zonule), skin and HDFs [21] using LC-MS/MS to identify a number of co-purifying associated proteins. Many of these were unique to their tissues of origin and mirrored the specific function these assemblies play in eye and skin.

**Fig. 7:**
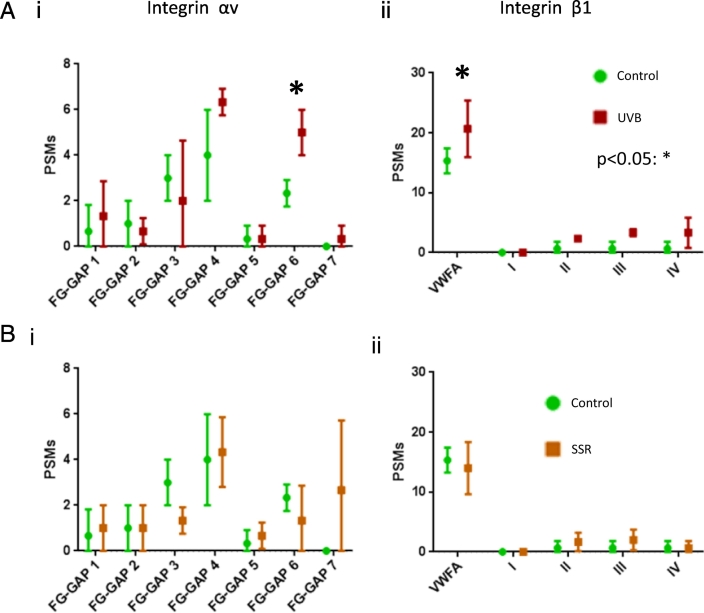
Broadband UVB irradiation of HDF-derived microfibril isolations may also lead to significant changes in the proteolytic susceptibility of co-purifying microfibril-associated receptors integrins αv and β1. LC-MS/MS-identified peptide sequences (PSMs: peptide prophet FDR ≤ 5%) in UVB-irradiated, SSR irradiated and control HDF-derived microfibril isolations were counted for each respective protein domain and normalised based on total spectrum count (N = 3; graphs = mean PSM count per protein domain and SD; statistical comparisons were made using Bonferroni-corrected multiple comparisons tests). Compared to control, broadband UVB-irradiation yielded significantly more PSMs at domain FG-GAP 6 (p = 0.0364) of integrin αv (Ai) and at the vWFA domain of integrin β1 (p = 0.023; Aii). SSR-irradiation however, failed to induce any significant changes in the PSM counts within domains of integrin αv (Bi) or integrin β1 (Bii).

In this experiment, a host of co-purifying proteins were detected within broadband UVB- and SSR-irradiated microfibril samples (Table S1). Of these, integrins αv and β1 which are ECM-cell signalling receptors for fibrillin microfibrils [95] (β1 for collagen VI microfibrils [96]) were also present. To test whether UVR may have also impacted on these key receptors, as with fibrillin-1 and COL6A3 (Figs. S2 and S3), PSMs identified in SSR-irradiated and broadband UVB-irradiated samples were mapped to the protein domain structures of integrins αv and β1. PSM counts per domain were compared to non-irradiated samples (Fig. 7). FG-GAP 6 domain for integrin αv (Fig. 7 Ai) and vWFA domain for integrin β1 (Fig. 7 Aii) were significantly different as a consequence of broadband UVB irradiation. SSR irradiation, however, did not lead to any significant, regional changes in proteolytic susceptibility within any of these proteins (Fig. 7 B). This is indicative once again that changes in regional susceptibility correlates with the energy of the incident irradiation (higher energy UVB was more damaging than SSR).

These results suggest that, in addition to core microfibril proteins fibrillin-1 and COL6A3, the tertiary structures of these networking receptor proteins may also be susceptible to UVR damage and consequently play a role in photoageing. It also demonstrates the potential of peptide fingerprinting for identifying damage not only within ECM assemblies but within receptors and possibly other species.

## Conclusion

Using LC-MS/MS peptide fingerprinting, we successfully identified broadband UVB- and SSR-damage within the fibrillin-1 monomer and COL6A3 chain of human fibrillin and collagen VI microfibrils. We effectively demonstrated that, although the quantity of damage to fibrillin-1 structure and to fibrillin microfibril ultrastructure correlates with the type of UVR exposure (broadband UVB or SSR), many of the foci of proteolytic susceptibility within the protein structure are conserved between UVR sources. Additionally, by comparing these to the COL6A3 monomer within co-purifying collagen VI microfibrils, we confirmed their comparative UVR-resistance on a monomeric level. However, we detected UVR-induced regional foci of increased proteolytic susceptibility within COL6A3 arising post-irradiation, indicating that this monomer is susceptible at least on a molecular scale. Furthermore, we showed that differences in chromophore content between fibrillin-1 and COL6A3 may account for the overall divergence in UVR susceptibility. We also demonstrated that, in addition to fibrillin-1 and COL6A3, the structures of co-purifying microfibril-associated receptor integrins αv and β1 are also susceptible to broadband UVB. Finally, this study highlights the use of LC-MS/MS peptide fingerprinting as an effective approach to measure post-translational damage on a molecular scale within ECM matrix assemblies and their constituent proteins.

The discoveries made in this study forward the fundamental understanding we have of the photodamaging effects of UVR to dermal proteins. The potential emerging from of this study is the identification of LC-MS/MS peptide mapping patterns within protein domains which were characteristic of fibrillin-1 and COL6A3 damage, shared between microfibrils separately irradiated with broadband UVB and SSR. Should these be detected within chronically photoaged microfibrils, they could serve as an early marker of the photoageing process.

In the skin photoageing field and that other chronic diseases (such as arthritis), protein-specific research tends to focus on deciphering mechanisms or evidence of damage through location (i.e. immunohistology) and relative quantification of abundance (i.e. Western blots and MS-based proteomics). However, due to the decades-long turnover of ECM assembles within these tissues, long-term accumulation of damage may not necessarily be reflective of protein location or abundance. These experiments indicate the potential of LC-MS/MS peptide mapping (fingerprinting) as a sensitive method of identifying underlying molecular damage on a historical level not only in photoageing but also in other chronic diseases. These methods could be employed on a whole tissue scale, where conventional, readily available, LC-MS/MS proteomic data-sets generated from diseased-state and normal tissues could be compared by mapping PSMs and data-dependently quantified peptide sequences to domain-level protein structure. This would allow the detection of damage to protein tertiary or quaternary structure, irrespective of whole protein-level fold change, traditionally measured.

## Materials and methods

### Study design

Fibrillin and collagen VI microfibrils were extracted and purified from cultured primary human dermal fibroblasts (N = 3; M79 [male aged 79 years], M75 and F75). Matched samples were split into three; one group was irradiated with a 30 J/cm^2^ dose of SSR (95% UVA, 5% UVB) [14], another with a 0.1 J/cm^2^ of broadband UVB [15] and the third was kept as control (unirradiated). Quantitative ultrastructural measurement of fibrillin microfibril bead morphology and periodicity (inter-bead distance) and of collagen VI microfibril periodicity was performed and compared using AFM. Fibrillin-1 and COL6A3 regional susceptibility to elastase digestion in response to UV irradiation was assessed by mapping the number of LC-MS/MS-detected PSMs and significantly different peptides (data-dependently quantified) to their respective protein domains. Regional differences were compared between UVR-irradiated (broadband UVB or SSR) and control, unirradiated.

### Reagents and cell acquisition

Chemicals were sourced from Sigma-Aldrich Co. Ltd. (Poole, UK) unless otherwise stated. This study was performed in accordance with the European Medicines Agency Note for Guidance on Good Clinical Practice and the Declaration of Helsinki 1964 (revised Seoul 2008). The use of human skin in this study was approved by North West Research Ethics Committee (ref# 14415). Donors gave written and informed consent prior to the collection of punch biopsies. Primary dermal fibroblasts were cultivated from skin biopsies taken from human donor photoprotected buttock. Incubations and cell cultures were conducted at 37 °C (5% CO_2_). Biopsies were incubated overnight in HBSS (Fisher Scientific, Loughborough, UK) containing 10% (v/v) PluriSTEM™ Dispase II solution. The dermis was separated from the epidermis using forceps, minced and further incubated in fibroblast DMEM media (Fisher Scientific, Loughborough, UK) containing 10% foetal calf serum (FCS), 1% amphotericin, 1% penicillin-streptomycin (Pen-Strep; Gibco, Paisley, UK) and 1% l-glutamine (all v/v). Fibroblast media was changed weekly until cells could be observed on sample plates.

### Microfibril extraction and purification

HDFs were cultured until confluent and maintained for a further five weeks in DMEM, high glucose, GlutaMAX™ Supplement (Fisher Scientific, Loughborough, UK) containing 10% (v/v) FCS and 50 μg/ml of Pen-Strep. Cells were then washed with phosphate buffered saline (PBS) within their culture flasks. Two milliliters of salt buffer (400 mM NaCl, 50 mM Tris-HCl and 10 mM CaCl_2_ at pH 7.4) containing 1 mg of bacterial collagenase IA and protease inhibitors: 0.01 mM phenylmethylsulfonyl fluoride (PMSF) and 0.03 mM N-ethylmaleimide (NEM) was then added directly to the culture flasks. These were digested on an orbital shaker for 2 h at room temperature.

Microfibrils were purified using size-exclusion chromatography via the ӒKTA Prime Plus Liquid Chromatography System (GE Healthcare; Little Chalfont, UK) as previously described [14,15,21]. After digestion with bacterial collagenase IA, HDF-derived mixtures were centrifuged at 5000*g* for 5 min. The supernatants were injected and ran at 0.5 ml/min within a column buffer (50 mM Tris-HCl and 400 mM NaCl at pH 7.4) through a GE HiScale 16/40 column containing Sepharose® Cl2B beads. Fractions containing both fibrillin and collagen VI microfibrils were collected based on spectrophotometric absorbance at 280 nm and were enriched in the void volume peak.

### Microfibril suspension UVR irradiation

Purified microfibril suspensions were split into three matched groups: control, broadband UVB-irradiated and SSR-irradiated. All suspensions (2 ml volumes) were irradiated within uncapped 35 mm × 10 mm polystyrene cell culture dishes (Corning, Flintshire, UK).

As previously described, SSR-designated suspensions were irradiated using a Solar Simulator (Applied Photophysics, Cambridge, UK) which is comprised of a xenon arc lamp light source fitted with a WG320 SSR filter (Schott, Stafford, UK) with a spectral output comprised of ~5% UVB and ~95% UVA (broad range wavelength output = 300–400 nm). A double grating spectroradiometer (Bentham Instruments Ltd., Reading, UK), calibrated to National Physical Laboratory (Teddington, UK) standards, was used to measure SSR spectral outputs [14]. Irradiance was measured using a UVX radiometer (UVR Products, Upland, CA, USA) fitted with a UVX36 full spectrum detector which was calibrated against the aforementioned spectroradiometer spectral output measurements. Suspensions were exposed to a dose of 30 J/cm^2^ (25 min and 30 s exposure time, irradiance = 19.6 mW/cm^2^) at a vertical distance of 20 cm from the source, at room temperature.

As previously described, broadband UVB-designated microfibril suspensions were irradiated with a broadband UVB spectrum using two 20 W Phillips TL-12 tubes (Eindhoven, The Netherlands) with an emission wavelength range of 270–380 nm (peak output of 313 nm) [15]. UVB waveband irradiance was measured using a UVX radiometer fitted with a UVX31 UVB detector calibrated using a double grating spectroradiometer to National Physical Laboratory standards. Suspensions were exposed to a dose of 0.1 J/cm^2^ (4 min 54 s exposure time, irradiance = 0.34 mW/cm^2^) at the centre of, and at a vertical distance of 16 cm from the source, at room temperature.

Aliquots of each sample were kept for ultrastructural analysis using AFM, and remaining suspensions were desalted in 0.22 μm filtered ultrapure water using Slide-A-Lyzer™ MINI Dialysis Devices (Thermo Fisher Scientific; Paisley, UK) for 4 h at 4 °C. Desalted samples were frozen at −80 °C and subsequently freeze-dried at −60 °C for 48 h. These were stored at −80 °C until their use in LC-MS/MS experiments.

### Microfibril atomic force microscopy

Glass coverslips were immersed in absolute ethanol overnight and then attached to magnetic AFM stubs using clear nail varnish. Microfibril suspensions were pipetted directly onto the coverslips and left for 1 min for the assemblies to adsorb to the glass surface. The majority of fluid was removed and the coverslip left to dry overnight. Coverslips were washed three times with ultrapure water to remove crystalized salt and left to dry prior to their use in AFM. As described previously, fibrillin and collagen VI microfibrils were scanned using peak force in Scan-Asyst® mode on a Multimode 8 atomic force microscope (Bruker; Billerica, MA, USA). Using new Scan-Asyst® Air tips (Bruker), fibrillin and collagen VI microfibrils were captured in a 2 × 2 μm scan area at a resolution of 512 pixels/line [21,37]. This produced a resolution of 3.9 nm/pixel, which was high enough for ultrastructural analysis as previously described [14,15,21].

WSxM v5.0 AFM Image Processing package [97] was used to digitally flatten each scan which were then exported in text image format. Height was subsequently corrected by subtracting negative background [21,98]. Images (41 pixels wide) of single, straightened fibrillin microfibrils were generated using the Straighten Curved Objects plugin in ImageJ (NIH; Bethesda, MA, USA) [99]. The image processing software: LFA, which was developed using Microsoft Visual Basic 6.0 by our group as described previously, was used to specify the height maxima of each fibrillin microfibril bead [21,44]. This generated 15 × 41 pixel snapshots of individual beads with the central pixel of the images corresponding to the centre of a single bead. Fibrillin microfibril bead height and morphology was measured and analysed using these snapshots. Fibrillin and collagen VI microfibril periodicity (inter-bead distance) was measured using the software package, Periodicity and Angles, developed by our group using Microsoft Visual Basic 6.0, as previously described [14,21,100]. A single measurement consisted of the distance from the centre of one bead to the centre of another.

### Microfibril fibrillin-1 and collagen VI alpha-3 peptide generation using elastase digestion prior to mass spectrometry

As described previously, the freeze dried microfibril purifications were re-suspended and denatured in 8 M urea [21]. Suspensions were then reduced by adding 10 mM dithiothreitol (DTT) for 30 min at room temperature and further alkylated using 50 mM iodoacetamide (IAM) in darkness, also for 30 min at room temperature. The suspension was then diluted to a concentration of 2 M urea using Tris-HCl buffer (100 mM Tris-HCl at pH 8.5), and porcine elastase (Sigma Catalogue # E1250) then added (2:1, enzyme:substrate ratio). Samples were digested at 37 °C for 4 h after which enzyme activity was then quenched using 5% (v/v) formic acid in ultrapure water. Peptide samples were then desalted using OLIGO R3 Reversed-Phase Resin beads (Thermo Fisher Scientific) and vacuum dried.

### Mass spectrometry

LC-MS/MS was performed by the Biological Mass Spectrometry Core Facility in the Faculty of Biology, Medicine and Health at the University of Manchester (Manchester, UK). As previously described in their protocols [21,101,102]: vacuum dried peptide samples were analysed via LC-MS/MS using an UltiMate® 3000 Rapid Separation LC (Dionex Corp; Sunnyvale, CA, USA) and an Orbitrap Elite mass spectrometer (Thermo Fisher Scientific). Peptide mixtures were separated using a 250 mm × 75 μm i.d. 1.7 mM BEH C18, analytical column (Waters, Hertfordshire, UK) on a gradient of 92% A (0.1% [v/v] formic acid in water) and 8% B (0.1% [v/v] formic acid in acetonitrile) to 33% B. These were run for 60 min with a flow rate of 300 nl/min. Peptides were automatically picked for fragmentation via data-dependent analysis.

### Mass spectrometry data analysis

The mass spectrometry proteomics data have been deposited to the ProteomeXchange Consortium via the PRIDE [103] partner repository with the dataset identifier PXD015149 and https://doi.org/10.6019/PXD015149.

For a detailed workflow summarising all the proteomic data analysis performed, please see Fig. S4. For peptide spectrum matching (PSM), mass spectra were extracted using extract_msn (Thermo Fisher Scientific). Mascot v2.5.1 (Matrix Science; London, UK) was used to correlate the spectra against the Swissprot_TreEMBL__2016_04 database (152,544 protein entries) [104] with the following search parameters: species - *Homo sapiens*; enzyme – non-specific; max missed cleavages – 1; fixed modifications - carbamidomethyl, mass – 57.02 Da, AA – C; variable modification – oxidation, mass – 15.99 Da, AA – M; peptide tolerance - 10 ppm (monoisotopic); fragment tolerance - 0.6 Da (monoisotopic). The PSMs reported were generated using Scaffold (Proteome Software; Portland, OR, USA). Only exclusive, unique peptide counts are reported for every dataset. FDR was calculated by Scaffold using protein and peptide probabilities assigned using the Trans-Proteomic Pipeline and the Peptide Prophet™ algorithm (Sourceforge; Seattle. WA, USA) [105]. Peptide Prophet FDR was thresholded to ≤5% for every dataset. The peptide sequence of each PSM was mapped to its respective protein domains of fibrillin-1 or COL6A3 (for an example, please see Fig. S5). The number of PSMs per domain was then counted for each sample. To allow comparisons across different samples (which unavoidably contain variations in protein abundance), the number of PSMs per domain were normalised across the whole experiment based on the total spectrum counts for fibrillin-1 or COL6A3 respectively [106]. Normalised PSMs per domain were then averaged across each group and subsequently heat mapped onto a domain schematic of fibrillin-1 or COL6A3 to show the average peptide yield within the different structural regions of these proteins. Bonferroni-corrected multiple comparisons tests between PSM counts per fibrillin-1 and COL6A3 domain were conducted using GraphPad Prism statistics software (GraphPad Software Incorporated; La Jolla, California, USA).

Data-dependent peptide quantification was performed using Progenesis QI (Nonlinear Dynamics, Waters; Newcastle, UK). Raw mass spectra files were imported and aligned using default settings. Data was then searched using Mascot v2.5.1 with same search parameters and on the same database as described earlier. This was then re-imported back into Progenesis QI where identified peptides and proteins were automatically matched. Raw abundance for each peptide was calculated by Progenesis QI as the sum of the each matched peptide ion abundance (individual peptide ion abundance defined as the sum of the intensities within the isotope boundaries) and normalised to a single run. Normalised peptide abundances were compared between matched samples (control vs. broadband UVB and control vs SSR) and a fold change for each peptide calculated automatically. Normalised peptide abundances compared between matched samples were statistically analysed within Progenesis QI using a paired ANOVA.

Peptide quantification data was exported from Progenesis QI to Excel (Microsoft Office, Microsoft, Manchester, UK). Only fibrillin-1 peptides which matched to Uniprot [104] accession number FBN1_HUMAN and COL6A3 peptides which matched to CO6A3_HUMAN, and which filtered to p ≤ 0.05, are reported. Significantly different fibrillin-1 and COL6A3 peptide sequences are reported alongside their fold changes, average normalised abundances per group and paired ANOVA p values.

The following are the supplementary data related to this article.

Supplementary figures Image 1

Table S1 List of proteins identified in all microfibril isolations using LC-MS/MS. Peptide spectrum matches are also reported for each protein (Peptide Prophet FDR ≤ 0.05). Table S1

## Funding disclosure

This study was funded by a programme grant from Walgreens Boots Alliance, Nottingham, UK.

## Declaration of competing interest

The authors declare that they have no conflicts of interest with the contents of this article. Walgreens Boots Alliance has approved this manuscript submission but exerted no editorial control over the content.
