# Supplementary material for: Proteomic fingerprints of damage in extracellular matrix assemblies
Source: Matrix Biol Plus. 2020 Jan 30;5:100027. doi: 10.1016/j.mbplus.2020.100027 (PMC7852314; doi:10.1016/j.mbplus.2020.100027)
Supplement: Supplementary file 1 — Supplementary figures [file mmc1.docx]

# Supporting Figures


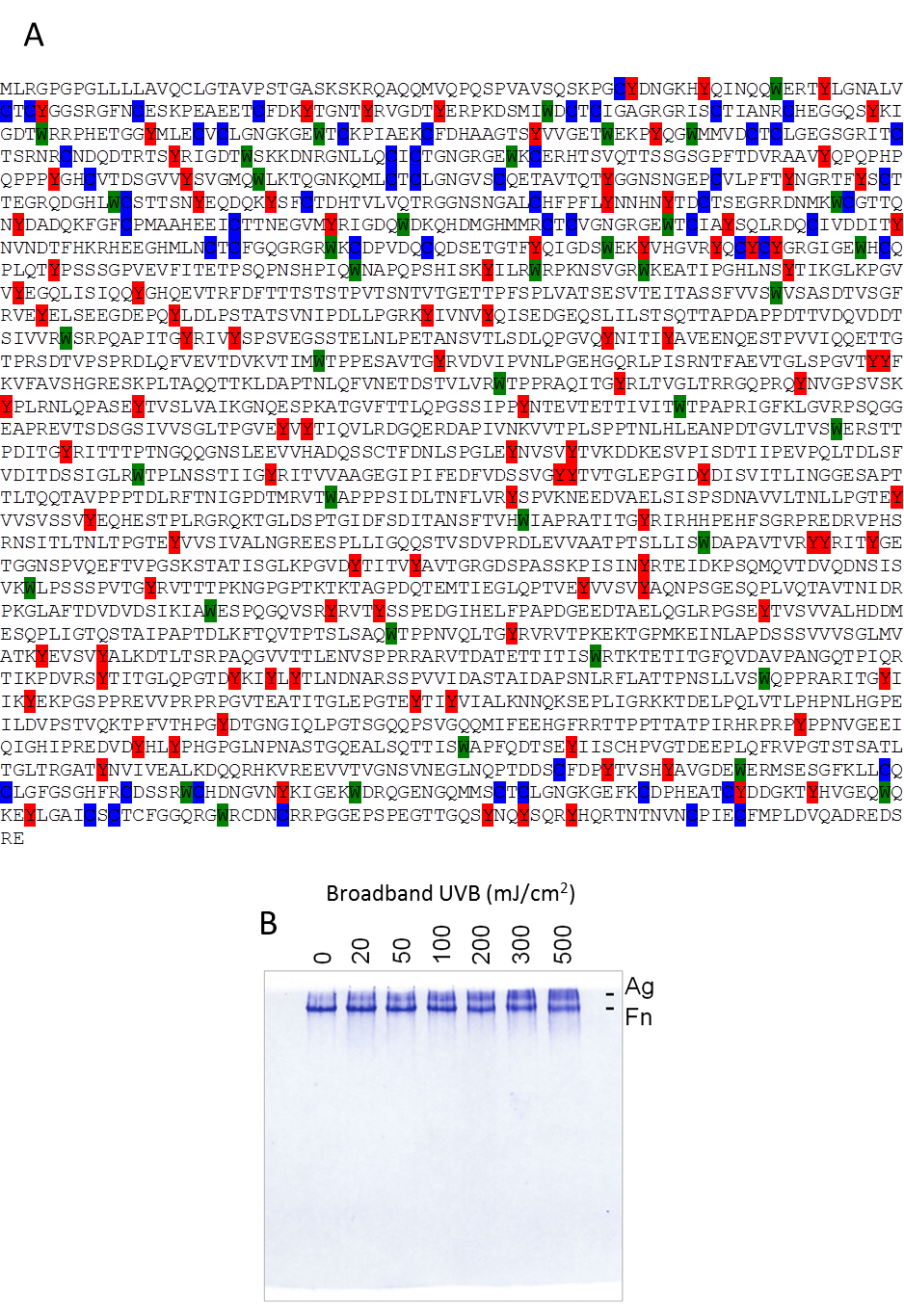


**Fig S1.** Despite the high presence of UVR-sensitive chromophores within the primary structure of fibronectin, exposure to high doses of broadband UVB fails to generate detectable protein fragmentation. The primary sequence of fibronectin **(A)** contains a number of UVR-sensitive chromophores spread throughout its structure (highlighted). Although both tryptophans (W, green) and tyrosines (Y, red) appear spread throughout, double-bonded cysteines (C, blue) are present only near the N-terminal and C-terminal ends of the protein. Theoretically, this suggests that a wide variety of degradation products may be generated, should UVR cleave the peptide bonds at these sites. Previously in 2010, we tested whether ascending doses of broadband UVB led to the detectable fragmentation of the fibronectin protein structure [15]. Using native gel electrophoresis, we showed that, even at the highest dose tested (500 mJ/cm^2^), broadband UVB failed to induce any observable fragmentation **(B)**, as seen by a lack of degradation products under the main fibronectin band (Fn). Instead, broadband UVB induced the dose-dependent aggregation (Ag) of protein above the main band. This suggests that cleavage of peptide bonds by UVR is minimal and instead, UVR-induced denaturation (cleavage of hydrogen and disulphide bonds) of the tertiary and quaternary structure may be the main mechanism behind protein damage. Native gel image **(B)** was reproduced from Supporting Figure S2 of the Sherratt *et al.* 2010 publication [15] with full copyright permissions obtained from publishers John Wiley & Sons Ltd.


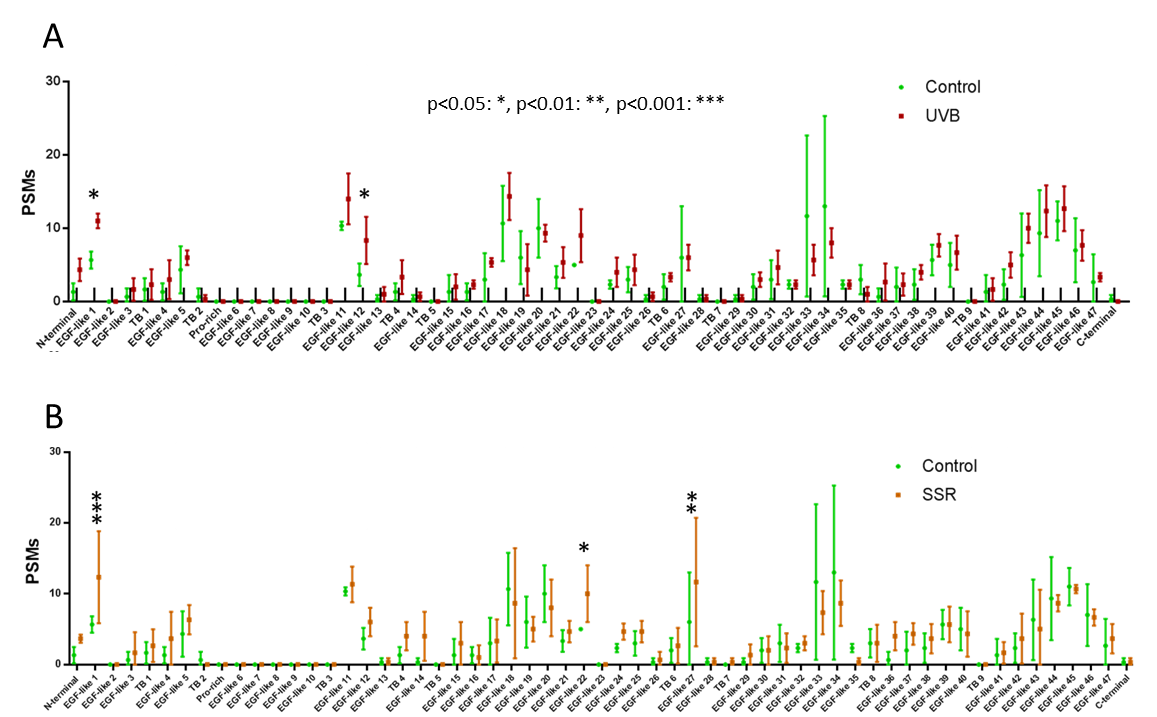


**Fig S2.** Broadband UVB and SSR irradiation of HDF-derived microfibril isolations leads to significant changes in the proteolytic susceptibility of specific protein domains within fibrillin-1. LC-MS/MS-identified fibrillin-1 peptide sequences (PSMs: peptide prophet FDR ≤ 5%) in UVB-irradiated, SSR irradiated and control HDF-derived microfibril isolations were counted for each respective protein domain and normalised based on total spectrum count (N=3; graphs = mean PSM count per fibrillin-1 domain and SD; statistical comparisons were made using Bonferroni-corrected multiple comparisons tests). Compared to control, broadband UVB-irradiated fibrillin microfibrils yielded significantly more fibrillin-1 PSMs at EGFs 1 (p=0.0112) and 12 (p=0.033) **(A)** and SSR-irradiated microfibrils yielded significantly more at EGFs 1 (p=0.0009), 22 (p=0.0195) and 27 (p=0.0063; **B**).


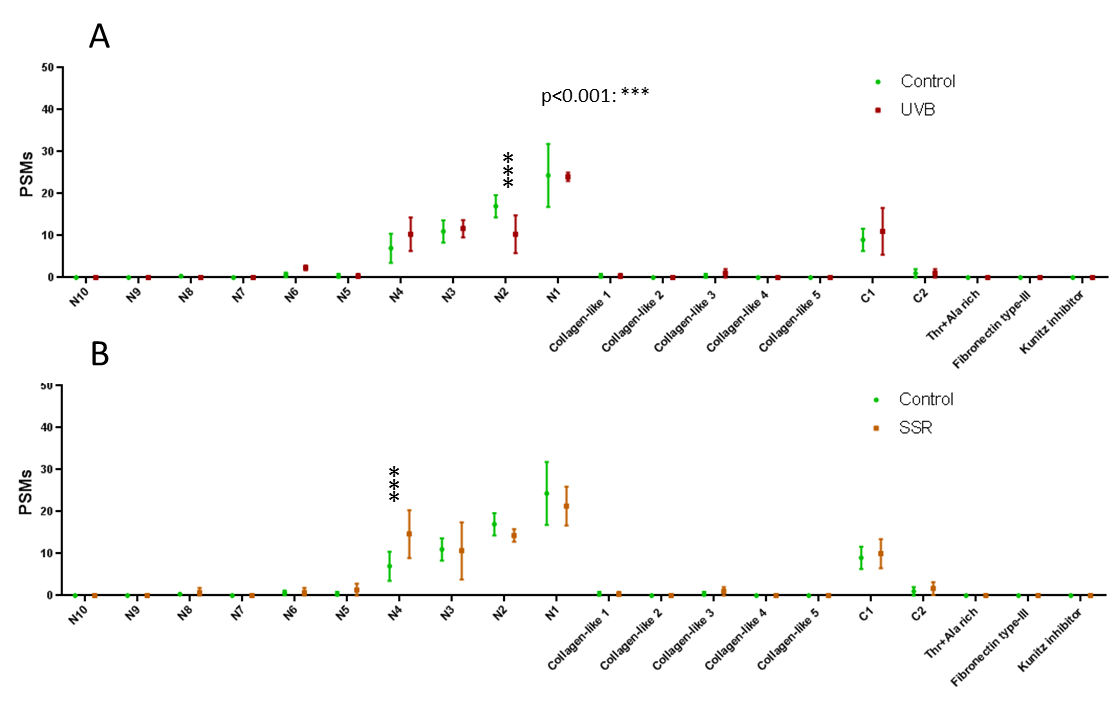


**Fig S3.** Broadband UVB and SSR irradiation of HDF-derived microfibril isolations also leads to significant changes in the proteolytic susceptibility of specific protein domains within COL6A3. LC-MS/MS-identified COL6A3 peptide sequences (PSMs: peptide prophet FDR ≤ 5%) in UVB-irradiated, SSR irradiated and control HDF-derived microfibril isolations were counted for each respective protein domain and normalised based on total spectrum count (N=3; graphs = mean PSM count per COL6A3 domain and SD; statistical comparisons were made using Bonferroni-corrected multiple comparisons tests). Compared to control, broadband UVB-irradiated collagen VI microfibrils yielded significantly less COL6A3 PSMs at vWA domain N2 (p=0.0004; **A**) whereas SSR-irradiated microfibrils yielded significantly more PSMs at vWA domain N4 (p=0003) **(B)**.


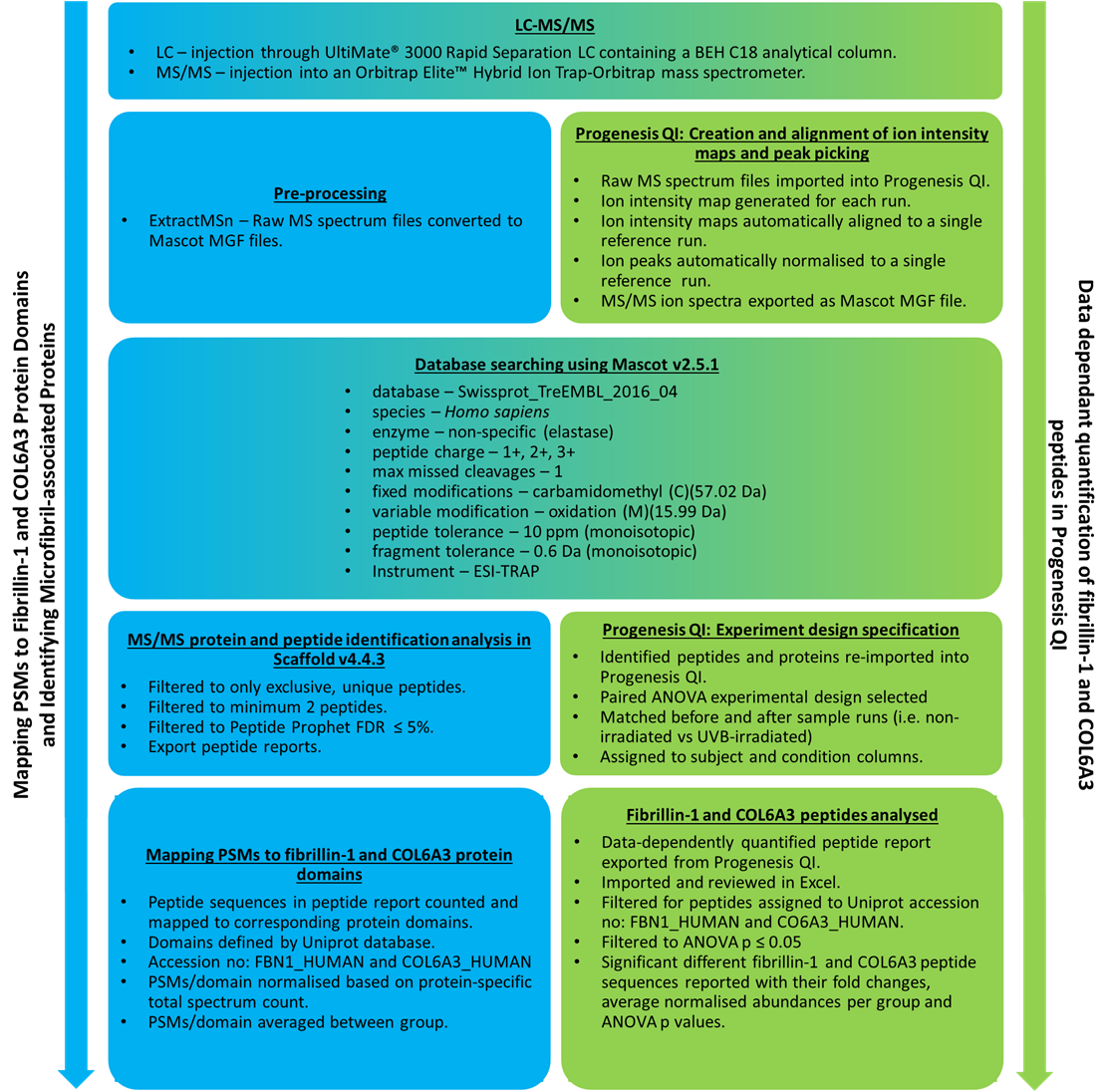


**Fig S4.** Work-flow summarising all the proteomic data analysis performed**.** The left blue path and associated blue text boxes detail the process of identifying and mapping peptide spectrum matches (PSMs) for fibrillin-1, COL6A3 and microfibril-associated proteins. The right green path and associated green text boxes details the process of data-dependent quantification of fibrillin-1 and COL6A3 peptides. Text boxes containing both blue and green colours detail steps pertinent to both processes.

**
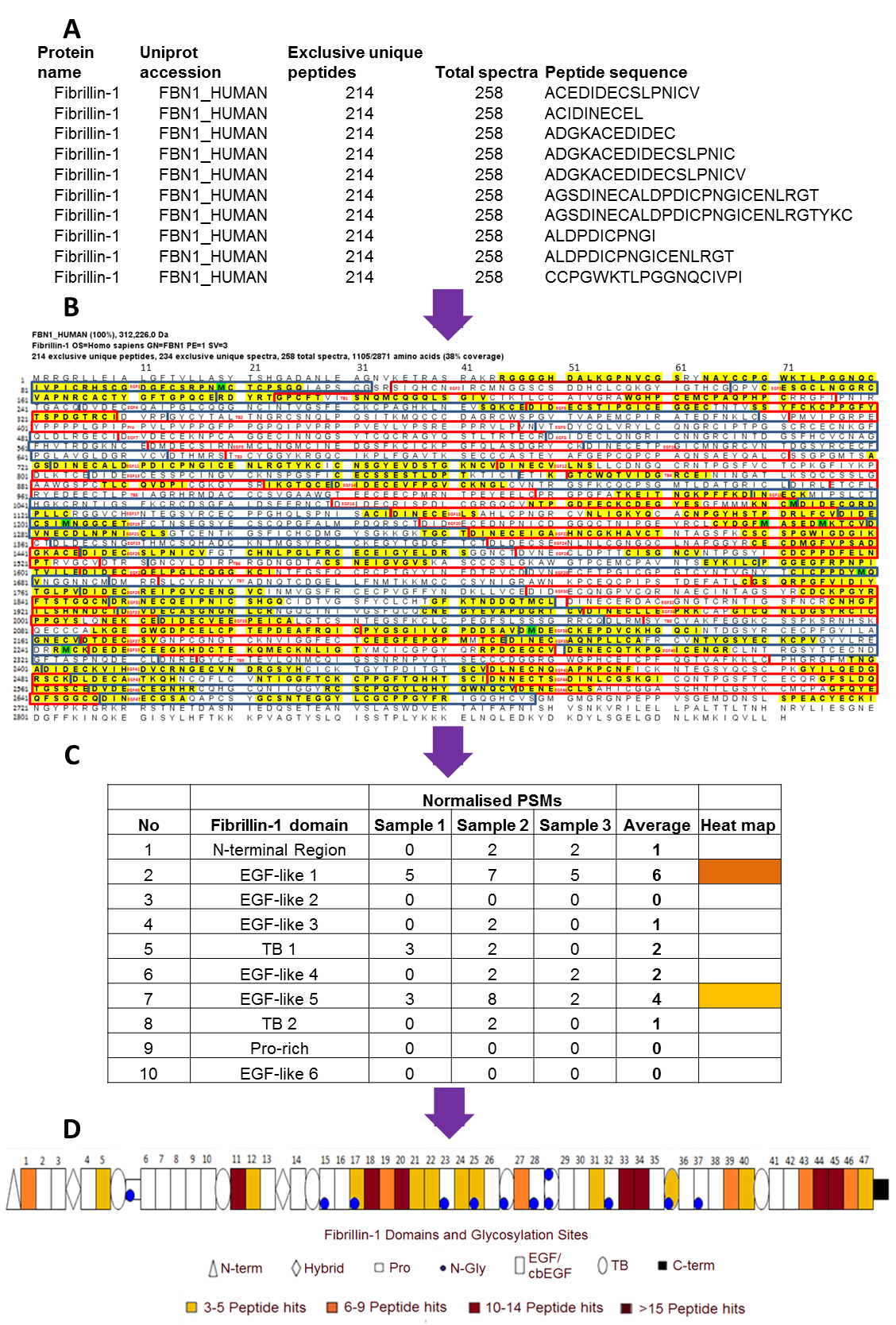
**

**Fig S5.** Example workflow showing the mapping of PSMs onto the domains of fibrillin-1. Fibrillin-1 peptides are identified in the peptide reports generated by Scaffold 4. The example table **(A)** depicts only the first ten fibrillin-1 peptide sequences identified out of 214 exclusive, unique peptides in this single sample. All 214 peptides are next mapped onto the primary sequence of fibrillin-1 (highlighted yellow) overlaid with its domain positions (sequences corresponding to each domain are boxed in alternating blue and red) **(B)**. The number of mapped peptides is then counted per domain. The process is repeated for all samples within the group, normalised based on the total spectrum counts across the whole experiment, and averaged across the group (example table **C** shows only the first ten fibrillin-1 domains) **(C)**. These are subsequently heat mapped onto a domain schematic of fibrillin-1 to show the average, regional yield of peptides across its structure **(D)**. Only domains containing an average of three peptides or more are shown.
